# Supplementary material for: Functionally and Metabolically Divergent Melanoma-Associated Macrophages Originate from Common Bone-Marrow Precursors
Source: Cancers (Basel). 2023 Jun 24;15(13):3330. doi: 10.3390/cancers15133330 (PMC10341323; doi:10.3390/cancers15133330)
Supplement: Supplementary file 1 [file cancers-15-03330-s001.zip › cancers-2459515-supplementary.pdf]

cancers MDPI

Supplementary Material

Communication

Functionally and Metabolically Divergent Melanoma-Associated Macrophages Originate from Common Bone-Marrow Precursors

Gabriela A. Pizzurro [1*], Kate Bridges [1], Xiaodong Jiang [2], Aurobind Vidyarthi [2], Kathryn Miller-Jensen [1,3] and Oscar R. Colegio [4,5]

cancers MDPI

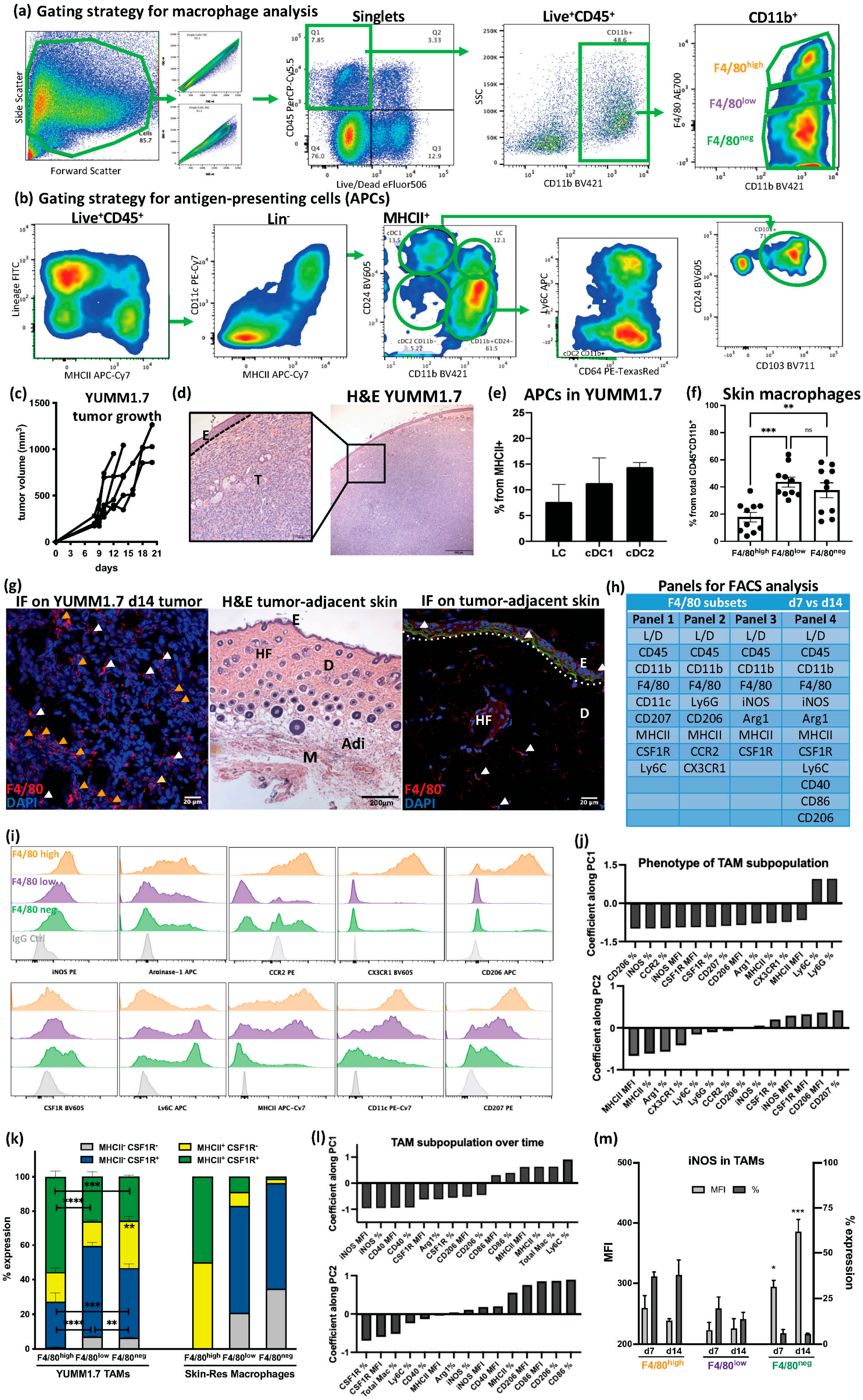

F4/80 subsets			d7 vs d14
Panel 1	Panel 2	Panel 3	Panel 4
L/D	L/D	L/D	L/D
CD45	CD45	CD45	CD45
CD11b	CD11b	CD11b	CD11b
F4/80	F4/80	F4/80	F4/80
CD11c	Ly6G	iNOS	iNOS
CD207	CD206	Arg1	Arg1
MHCII	MHCII	MHCII	MHCII
CSF1R	CCR2	CSF1R	CSF1R
Ly6C	CX3CR1		Ly6C
			CD40
			CD86
			CD206

cancers MDPI

Supplementary Figure S1. Tumor-associated macrophage subsets in the i.d.-injected YUMM mouse melanoma model. **(a)** Flow cytometry gating strategy for analyzing F4/80 macrophage subpopulations in YUMM1.7 tumors. **(b)** Flow cytometry gating strategy for determining APC subsets in YUMM1.7 tumors. **(c)** Individual tumor growth curves of the YUMM1.7 cell line in C57Bl/6J. **(d)** H&E and detail from i.d. YUMM1.7 tumor at day 14, injected in Myb mice. E = epidermis, T = tumor. **(e)** FACS quantification of skin myeloid APC subsets in YUMM1.7 tumors in Myb mice. n=4. **(f)** Skin-resident macrophages determined by their F4/80 expression in C57Bl/6J. n=10, pooled from at least 3 independent experiments. **(g)** Immunofluorescence (IF) for F4/80 in YUMM1.7 melanoma tumors. As a reference, H&E and IF in tumor-adjacent skin. D=dermis, E=epidermis, HF=hair follicle, M=muscle, Adi=adipose tissue. White triangles indicate single F4/80^{+} cells, while orange triangles point out clusters of TAMs. **(h)** Marker panels used for phenotyping myeloid cells infiltrating YUMM1.7 tumors and normal skin. **(i)** Representative histograms of flow cytometry marker expression analyzed in the TAM subset comparisons. **(j)** Coefficients along PC1 (top) and PC2 (bottom) for the PCA of phenotypic markers of F4/80 subpopulations in tumor vs skin. As indicated, either percentage of marker expression (%) or % and MFI were included in the analysis. **(k)** Analysis of monocyte-to-macrophage marker co-expression (CSF1R and MHCII) in TAM subpopulations from YUMM1.7 tumors, and a control skin. Statistical analysis showing comparisons between marker-expressing subsets within TAMs. **(l)** Coefficients along PC1 (top) and PC2 (bottom) for the PCA of phenotypic markers of F4/80 subpopulations in tumors day 7 vs day 14. As indicated, either percentage of marker expression (%) or % and MFI were included in the analysis. **(m)** Detail of the evolution of percentage and MFI of M1-marker iNOS expression in F4/80 TAM subsets. n=5 mice per TAM group, pooled from 3 independent experiments. ns= not significant, *p<0.05, **p<0.01, ***p<0.001.

cancers

MDPI

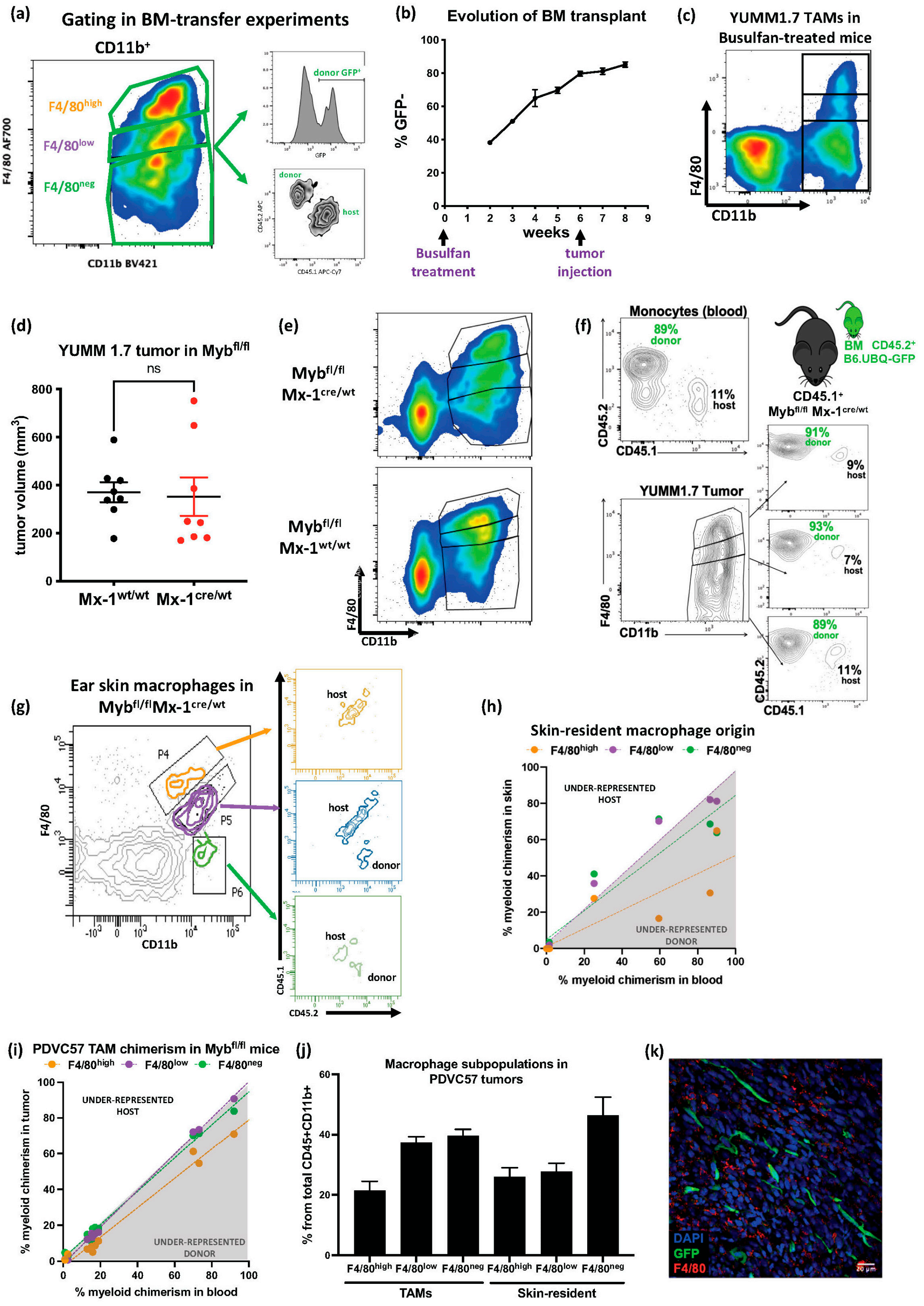

cancers MDPI

Supplementary Figure S2. Origin of melanoma-associated macrophage subsets**. (a)** Flow cytometry strategy for determining TAM origin in YUMM1.7 tumors. Fate-mapping of BM-derived cells was identified by CD45.1/CD45.2 expression and validated by GFP^{+}. **(b)** Assessment of BM engraftment after Busulfan treatment and transplant. Blood samples were extracted and analyzed weekly. The graph shows the percentage of GFP^{-} cells from total $CD45^{+}$ cells in GFP^{+} mice. n=5. **(c)** Representative plot of TAM subpopulations in Busulfan-treated mice 8 weeks after transplant. **(d)** YUMM1.7 tumor size in $Myb^{fl/fl}$ mice at day 14, at the time of processing for analysis. **(e)** Representative flow cytometry plots of TAM subpopulations in YUMM1.7 tumors in $Myb^{fl/fl}Mx\text{-}1^{cre/wt}$ and $Myb^{fl/fl}Mx\text{-}1^{wt/wt}$ mice. **(f)** Schematic of the analysis of macrophage origin in tumor samples in $Myb^{fl/fl}Mx\text{-}1^{cre/wt}$ mice, and comparison with blood monocyte chimerism. Host immune cells are $CD45.1^{+}GFP^{-}$ and donor cells are $CD45.2^{+}$ and GFP^{+}. **(g)** Representative plots of the analysis of the origin of macrophages in ear skin from BM-transplanted Myb mice. **(h)** Assessment of macrophage origin in skin of tumor-bearing $Myb^{fl/fl}Mx\text{-}1^{cre/wt}$ and $Myb^{fl/fl}Mx\text{-}1^{wt/wt}$mice. Linear regressions were performed to compare the chimerism of myeloid cells observed in circulation and the chimerism of myeloid tumor-infiltrating cells. Skin samples, n=8, pooled from 2 independent experiments. **(i)** Assessment of macrophage origin in PDVC57 tumors in $Myb^{fl/fl}Mx\text{-}1^{cre/wt}$ and $Myb^{fl/fl}Mx\text{-}1^{wt/wt}$ mice. Linear regressions were performed to compare the chimerism of myeloid cells observed in circulation and the chimerism of myeloid tumor-infiltrating cells. TAM subsets were analyzed separately. Tumor samples, n=10, pooled from 2 independent experiments. **(j)** Quantification of TAMs and skin-resident macrophage subsets based on F4/80 expression in i.d.-injected PDVC57 tumors in C57Bl/6J. n=6-10, pooled from 2 independent experiments. **(k)** Immunofluorescence staining on YUMM1.7 tumor from Busulfan-treated mice, showing only GFP^{+} stromal cells.

cancers

MDPI

(a)

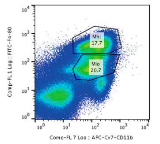

Sample_1	F4/80^high	161416
	F4/80^low	467320
Sample_2	F4/80^high	522565
	F4/80^low	1471916
Sample_3	F4/80^high	218228
	F4/80^low	624604

(b)

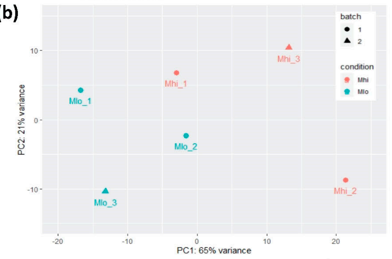

(c)

F4/80^high TAMs

	Log2FoldChange	p-value (adj)
Gm28049	6.584604715	0.009240763
St18	5.615315739	0.047023634
Gbgt1	3.83766266	0.000686275
Mrvi1	3.799213555	0.022372385
Gm6710	3.400979485	0.042578028
RP23-26P10.1	3.304597769	0.00095194
Cd163	3.152898245	0.02167177
Slco2b1	3.004562616	0.005421037
Rapgef5	2.936676966	0.023225688
Gm15880	2.810271781	0.022372385
Ms4a14	2.621480237	0.000211194
Scamp5	2.468269971	0.000584185
Spin4	2.398096625	0.029308747
Zdhhc14	2.387773517	0.014463673
Hpgds	2.372577924	0.004765114
Lonrf3	2.305200623	0.020415803
Apold1	2.233013864	0.029308747
Mrc1	2.219736182	0.010974313
Itga9	2.208618885	0.005793151
Igf1	2.163549432	0.007743335
Gm10134	2.152394047	0.001137803
Tdrkh	2.017959613	0.010629957
C1qa	1.960986541	0.047382687
Gpr34	1.948690168	0.005307293
Ms4a7	1.935309663	0.024302447
Arhgap19	1.931112894	0.00145479
Cadm1	1.907844223	0.011228814
Olfml3	1.899902242	0.002047071
Stab1	1.832088096	0.034524385
C4b	1.772663925	0.01413023
Blnk	1.770030187	0.021973892
Prune2	1.704464226	0.020415803
Mertk	1.665602583	0.022509876
Gdpd5	1.646956437	0.022509876
Gm37795	1.618343355	0.030222198
Bbs7	1.616514749	0.036947432
Abcc3	1.61598911	0.038339054
Ccl8	1.601805093	0.030222198
Frmd4b	1.57734899	0.000506847
Rab3il1	1.545887596	0.035665271
Ctla2b	1.503755412	0.019820169
Cd38	1.468646345	0.027659319
RP24-180F7.2	1.46376072	0.014963899
Sepp1	1.458027668	0.02395971
Abcb1b	1.361607969	0.001137803
Zmynd15	1.302103388	0.049109972
Pltp	1.278872876	0.041641002
Pdgfc	1.214844511	0.042578028
Cebpa	1.1662205	0.012421469
Maml2	1.078892543	0.022372385
Cd72	1.055101448	0.042578028

F4/80^low TAMs

	log2FoldChange	p-value (adj)
Sytl4	-7.847994864	0.000502805
Grm1	-7.463531415	0.007658365
Afap1l2	-7.340588703	0.000584185
Sh3tc2	-6.159471225	0.013968525
Gm37334	-5.309857158	4.75E-05
Abca6	-5.239063108	0.00572596
Cubn	-5.204547692	0.011228814
6230415J03Rik	-5.180191644	0.004088306
Gm37873	-5.126793775	0.023225688
Cttnbp2	-5.07996975	4.75E-05
AW121686	-4.920024497	0.022372385
Gm37115	-4.830402755	0.000146079
Epha4	-4.787700318	0.002253627
BC039771	-3.94560155	0.023687276
Grm6	-3.867535917	0.008313685
Btnl9	-3.800560949	0.032456106
Ccdc64b	-3.725221462	0.004369468
Ttc26	-3.636662517	0.002592003
Arhgef28	-3.610244951	0.004918731
Gm10282	-3.418129862	0.047382687
Ighg1	-3.244328781	0.000130998
Gm7292	-2.922087549	0.032008481
Dcstamp	-2.750728894	0.002592003
Gm29666	-2.744594656	0.047398534
Bmx	-2.427075833	0.039614528
Ctcflos	-2.424551074	0.001616943
Mcmdc2	-2.38201597	0.048562434
Gm15513	-2.378260731	0.018447742
Cldn15	-2.374790715	0.042635601
Pbld2	-2.274623258	0.017163329
Gm37760	-2.15590194	0.045151748
Smpd3	-2.138543983	0.000584185
Abcc2	-2.063266298	0.012898923
Gm28187	-2.044480862	0.009307088
Dach1	-1.962524055	0.023687276
Ffar2	-1.96203876	0.004918731
Zbtb16	-1.931605511	0.026998616
Nxpe4	-1.917520391	6.43E-06
I830127L07Rik	-1.884296572	0.002253627
Prg2	-1.869431428	0.001137803
Slco4c1	-1.81998291	0.00095194
Chil3	-1.806328695	0.000502805
Sag	-1.751140706	0.047023634
Vcan	-1.751118976	0.018851697
Mylk3	-1.732723562	0.047382687
Ear2	-1.718321127	0.025561278
Gata1	-1.662998314	0.036938585
Cd177	-1.637362825	0.024302447
Ccr3	-1.635771299	0.006122297
Ifitm6	-1.627062142	0.002253627
Col27a1	-1.618677663	0.040805213

(d)

F4/80^high TAMs

NAME	SIZE	NES	NOM p-val	FDR q-val
HALLMARK_E2F_TARGETS	192	2.464	0.000	0.000
HALLMARK_MYC_TARGETS_V1	191	2.448	0.000	0.000
HALLMARK_MYC_TARGETS_V2	58	2.218	0.000	0.000
HALLMARK_G2M_CHECKPOINT	182	1.772	0.000	0.003
HALLMARK_OXIDATIVE_PHOSPHORYLATION	194	1.551	0.000	0.020
HALLMARK_PROTEIN_SECRETION	91	1.290	0.074	0.175
HALLMARK_ALLOGRAFT_REJECTION	185	1.285	0.058	0.159
HALLMARK_PEROXISOME	92	1.250	0.090	0.182

F4/80^low TAMs

NAME	SIZE	NES	NOM p-val	FDR q-val
HALLMARK_INTERFERON_ALPHA_RESPONSE	90	2.146	0.000	0.000
HALLMARK_INTERFERON_GAMMA_RESPONSE	183	2.096	0.000	0.000
HALLMARK_TNFA_SIGNALING_VIA_NFKB	191	2.067	0.000	0.000
HALLMARK_EPITHELIAL_MESENCHYMAL_TRANSITION	180	1.958	0.000	0.000
HALLMARK_HYPOXIA	178	1.881	0.000	0.001
HALLMARK_COAGULATION	104	1.847	0.000	0.001
HALLMARK_INFLAMMATORY_RESPONSE	185	1.776	0.000	0.004
HALLMARK_UV_RESPONSE_DN	137	1.714	0.000	0.006
HALLMARK_REACTIVE_OXIGEN_SPECIES_PATHWAY	46	1.696	0.007	0.006
HALLMARK_APOPTOSIS	156	1.666	0.000	0.006
HALLMARK_APICAL_JUNCTION	166	1.578	0.002	0.012
HALLMARK_MYOGENESIS	163	1.562	0.000	0.013
HALLMARK_ESTROGEN_RESPONSE_LATE	163	1.561	0.000	0.012
HALLMARK_ANGIOGENESIS	34	1.481	0.028	0.026
HALLMARK_ESTROGEN_RESPONSE_EARLY	171	1.379	0.013	0.067
HALLMARK_KRAS_SIGNALING_UP	176	1.376	0.011	0.064
HALLMARK_COMPLEMENT	171	1.351	0.026	0.076

(e)

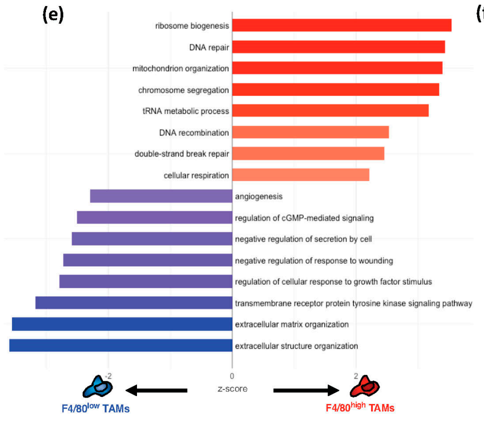

(f)

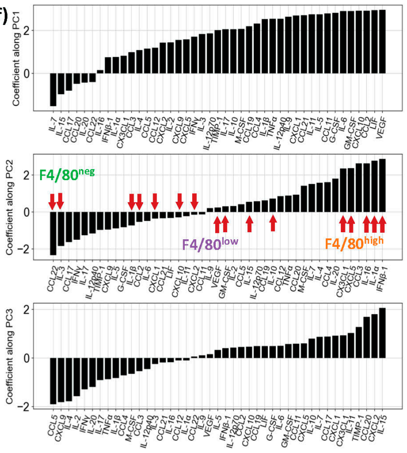

(g)

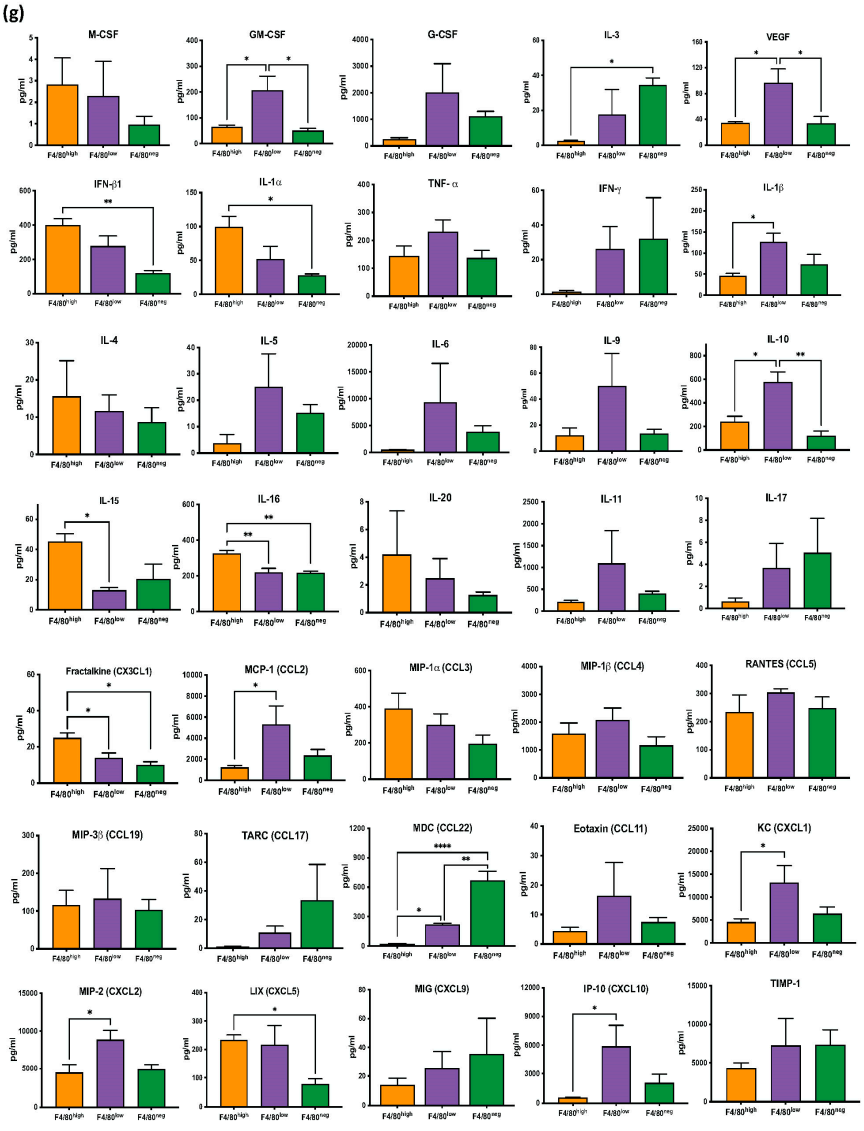

Supplementary Figure S3. Gene expression profiling and functional analysis of TAM subsets in YUMM1.7 tumors. **(a)** Representative plot of sorted F4/80^{+} TAMs. Total number of cells sorted from TAM subsets for bulk RNAseq. **(b)** PCA embedding of bulk RNAseq samples after batch-correction. **(c)** Top 50 differentially-expressed genes (DEGs) for each F4/80 subset (p-adj < 0.05, abs(log2FC) > 1). **(d)** GSEA results showing significantly upregulated Hallmark gene sets from the mouse MSigDB, with a FDR<25% for each TAM subset. **(e)** Enrichment of F4/80^{high} (right, red) and F4/80^{low} (left, blue) DEGs (protein-coding genes only) for gene ontology (GO) biological process terms. Bar lengths represent associated z-score. **(f)** Coefficients along PC1 (top), PC2 (middle), and PC3 (bottom) for the PCA of multiplex protein secretion assay performed on sorted F4/80^{high}, F4/80^{low} and F4/80^{neg} subsets from YUMM1.7 samples. Along PC2, samples separated and clustered by F4/80 expression level. Red arrows indicate the proteins that showed significantly different levels of secretion, detailed in (g). **(g)** Individual bar graphs of protein secretion from sorted TAM subsets which showed significant differences or trends between them, with relevance to immune function in the TME. *p<0.05, **p<0.01, ****p<0.0001.
